# Integral health care for transgender adolescents: subsidies for nursing practice

**DOI:** 10.1590/1518-8345.6276.3810

**Published:** 2022-11-25

**Authors:** Paula Daniella de Abreu, Pedro Fredemir Palha, Rubia Laine de Paula Andrade, Sandra Aparecida de Almeida, Jordana de Almeida Nogueira, Aline Aparecida Monroe

**Affiliations:** 1 Universidade de São Paulo, Escola de Enfermagem de Ribeirão Preto, PAHO/WHO Collaborating Centre for Nursing Research Development, Ribeirão Preto, SP, Brazil; 3 Universidade Federal da Paraíba, Centro de Ciências da Saúde, João Pessoa, PB, Brazil

**Keywords:** Nursing, Social Networking, Transgender Persons, Gender Identity, Family, Public Health

## Abstract

**Objective:**

to analyze the integral health care for transgender adolescents from the perspective of their guardians.

**Method:**

qualitative research based on the Social Network framework proposed by Lia Sanicola, developed with 22 guardians of transgender adolescents in Brazil through semi-structured individual online interviews. The empirical material was analyzed using the content analysis technique, thematic modality.

**Results:**

lack of ambience was observed, in addition to technical unpreparedness of health professionals in relation to the theme at all levels of care, transphobia, centralization of care in scarce qualified services for transgender children and youth, absence of family support, lack of health promotion actions within the community, especially in the school environment, and the common support from non-governmental initiatives.

**Conclusion:**

the centralization of actions in scarce specialized services in the country, and the structural transphobia can compromise the integral health care for transgender adolescents. There is an urgent need for a network of care capable of assisting the joint action by multi and interdisciplinary teams, with greater proactivity of the nurse with the transgender adolescent and their guardians in individual and collective actions; ambience; health promotion in schools for visibility and support in Primary Health Care since childhood.

## Introduction

Transgender adolescents and their guardians experience challenges in terms of visibility in the cis-normative context that makes them vulnerable. Thus, these people require support networks and health policies that are structured in a protective model in the field of integral health. The approach to transgenderism, although protected by legislation in most countries, is incipient in the health context on a world stage^1^ ^-^ ^4^.

Adolescences, in a plural form, compose the universe of emancipatory experiences between childhood and adulthood, which are associated with singular characteristics of the individual and intersectionalities of social, racial, ethnic, and spiritual segments, in addition to gender identity and sexual orientation. These experiences are influenced by the historical and multicultural origin, and by social determination^5^.

Although the right to integrated health actions for adolescents is provided since the 1988 Brazilian Federal Constitution (FC) and creation of the Unified Health System (SUS)^6^ ^-^ ^7^, in addition to presenting the Child and Adolescent Statute (ECA) as a main normative instrument of their rights to life and health, as well as to expression, support by an interprofessional team, preservation of autonomy, identity, and protection^8^, adolescence in the transgender context has challenges to be faced, due to stigma^9^ ^)^ and the double vulnerability of being an adolescent and a trans person, immersed in structural transphobia, potentiated at the intersection of oppressed and discriminated social identities.

In the context of health, the bond between professionals and transgender people, especially adolescents, should begin from the projection of the welcoming look and legitimization of the name chosen by the trans person, anchored to affective, social, and political aspects^10^. However, even though the “social name’’ is a right instituted by the SUS, since 2006, through the Letter of the Health Users Rights^11^ ^)^ and, subsequently, composes the field for registration in the National Health Card (SUS Card)^12^, in the practice of health services, this right is commonly violated and denied, which implies violence and potential rupture of bonds^10^. Besides these aspects, transgender adolescents also face the bureaucratic barriers to name rectification, since it occurs upon judicial authorization^13^ in the case of underage people, with disrespect for the name and treatment pronouns being difficulties experienced in all social contexts, including the nuclear family and school^9^.

In addition to the National Guidelines for Integral Health Care for Adolescents and Youth in the Promotion, Protection, and Recovery of Health^5^, the National Policy of Integral Health for Lesbians, Gays, Bisexuals, *Travestis*, and Transsexuals (LGBTI+), was instituted by the Ministry of Health through Ordinance no. 2,836, of December 1, 2011, which includes the responsibility articulated between federative entities in the strategies that promote integral health care for transgender adolescents, based on acceptance and support^14^.

The SUS transsexualization process also became an important milestone in accessing health care with the expansion of assistance for transgender men and *travestis* ^15^ ^-^ ^16^. In spite of advancements in the field of access rights, structural transphobia marks the distance of transgender people from the specific care they seek. Barriers include the denial of their identity and name; and difficulty in accessing services in the Health Care Network (HCN), especially due to the unpreparedness of health professionals when providing care. This scenario can be intensified in private services in relation to the SUS, and can result in indiscriminate self-hormonization alternatives^10^.

In adolescence, the feeling of body modification can intensify and be related to issues of self-acceptance in dealing with the body with which they do not identify, especially due to the discomfort with secondary sex characteristics. Most trans adolescents express a desire for hormones^9^, but it is important to highlight that the transgender identity is independent of any physical modification, and, in this sense, the nurse needs to be able to listen to the needs required by the adolescent that may or may not be associated with issues of transgenderism, or even be transversal to it, as well as the potential risks caused by self-promoted hormonization, psychological pain, and suicide risk^9^.

Regarding the care of transgender children and adolescents, Resolution no. 2,265, of September 20, 2019, provides for assistance through specialties that meet the needs of the Singular Therapeutic Project (STP). In Pre-puberty, it is recommended follow-ups by the team; in Puberty, there is the possibility of hormonal blockade to prevent the emergence of secondary sex characteristics, upon the consent of the team and legal guardians; and, from the age of 16, there is the possibility of cross-hormonization for feminization or masculinization, upon consent. From the age of 18, surgeries are provided, including metoidioplasty, which is no longer experimental^17^.

In this sense, the consolidation of the SUS reverberated significantly on the structuring of health policies aiming for well-being and equity. Nevertheless, the dynamic political scenario and cis-normative model need to be critically analyzed in the historical and cultural contexts of advancements and setbacks in guaranteeing the rights of trans people^5^. Concerning the interlocution with the rights of transgender adolescents, important challenges are observed, since they are daily violated by negligence and necro-trans-politics which naturalizes the precariousness of trans life in a country that leads the number of murders, and limits the life projects of trans adolescence, as well as the life course and aging of this population^1^.

The guarantee of the right to health for trans adolescents is anchored not only in protocols and access to health services themselves, as it demands the effective practice of trans citizenship, that is, trans protagonism in social and health spaces, which requires from health professionals^18^, especially the nurse, skills to embrace and enable trans participation in the health care production process.

Health professionals, especially those in Nursing who comprise Primary Health Care (PHC) can establish a link and benchmark in the recognition of the support network for transgender adolescents and their families, in order to consider their social context, and, according to that, meet their needs, in a socio-historical and cultural perspective, and not only biological, enabling the guarantee of the rights and consolidation of policies for the integral health care in HCN^19^.

The analysis of integral health care in the context of trans adolescence, in the light of the dynamics of the social network, reflects necessary nuances to discuss the role of Nursing and its potentialities with the health team. Social networks can be defined as a web of ties established between the person and the primary network: family, relatives, friends, and neighbors, in addition to the relationship with the secondary network: ties established with institutions, market organizations, and the third sector^20^.

The identification of gaps for the access to integral health care for trans adolescents requires listening to them and also to the actors who comprise their social network, since the protection and management of care is guided by different fronts, with the family as the first network of socialization, recognition, and responsibility for the care of the transgender person since childhood, thus, a potential network of support or violence, and lack of protection for the trans adolescent. Listening to mothers, fathers, and guardians can complement the understanding of the challenges to be faced by both, as well as possible ways for structuring acceptance and empowerment in the sense of supporting their transgender children. The perspective of these guardians needs to be analyzed and contextualized to the role of the nurse while social actor inserted in a support network, as the encounter between professionals and patients comprises the dimension of care in the territory of micropolitics in health, inserted in the planning with the multi/interdisciplinary team, in the production of a network of care, in which social and health policies operate under the influence of the State and civil society in the dispute over projects that will impact health living conditions^21^.

This study was motivated by the following guiding questions: What are the experiences of mothers, fathers, or guardians of transgender adolescents in the context of health? How does the integral health care for transgender adolescents occur from the perspective of the guardians? What is the role of Nursing in the integral health care for transgender adolescents? Thus, the study aimed to analyze the integral health care for transgender adolescents from the perspective of their guardians.

## Method

### Study design

Qualitative research, guided by orientations of the Consolidated Criteria for Reporting Qualitative Research (COREQ)^22^, and based on the theoretical and methodological framework of Social Network proposed by Lia Sanicola, which deals with the dynamics of social network relationships, and is configured in an interaction network exercised according to a certain central object, having functions of support or containment and control. These networks can contribute to the strengthening of emotional, on-site, instrumental, and informational support, as well as self-support, and also provide changes from the status of dependence to that of autonomy for the target group^20^.

### Study scenario

The study comprised the 27 Federative Units of Brazil, and was supported by referral outpatient clinics, Municipal Health Departments, associations, and peer groups active in the theme with representatives in all Brazilian states, namely: National Level: LGBT National Alliance, Brazilian NGO Mothers for Diversity, Association of Crossdressers and Transsexuals of Brazil; State Level: Trans Care and Welcoming Space - Clinics Hospital/Pernambuco; Municipal Level: Center for Integral Health Care for the Black and LGBT Population/Jaboatão dos Guararapes, and Health Care Outpatient Clinic for Trans people in Porto Alegre.

Access to these places began with the insertion of the researcher in welcoming environments for transgender people at the municipal level, which indicated the other places at the state and national levels in a progressive way. Although most of these places focus on trans adults, the contribution was made with the support for research and indication of specific non-governmental representations of peer groups coordinated by mothers of LGBTIA+ people, and composed of guardians of transgender children and adolescents.

After contacting theses representations, the researcher sought to establish a bond with professionals and representatives of these spaces, participating in meetings and presenting the study, which culminated in the conformation of a network that shared the scheduling form for participation in the research. The document was created using Google Forms, and directed to eligible participants and indicated by the members of the network.

It is important to emphasize that the national peer group, specific for guardians of transgender children and adolescents does not allow the insertion of researchers, and demanded the construction of a bond and trust to share the scheduling form, followed by the sharing of the initial participants among between groups with consensus and certification of the research importance. Other spaces did not integrate the study because their activities were suspended during the period of the COVID-19 pandemic, and because of sufficient sampling in a specific national group.

### Period

The production of empirical data occurred between August and October, 2021.

### Population

The study was composed of 22 participants, including mothers, fathers, or guardians of transgender adolescents, who were willing to giver their testimonies. People aged 10 to 19 were considered as ‘’adolescents’’^5^. The sampling process followed the recommendations of qualitative research, which considers the set of characteristics belonging to a clear social group in order to privilege the specificities. The ideal qualitative sample thoroughly reflects the phenomenon, therefore, this study did not seek generalizations and numerical criteria, but the constant analysis of homogeneity, diversity, and intensity of information^23^.

The recruitment of participants in studies involving social taboos may generate discomfort, thus, the pause criterion demanded constant analysis of the speeches after each interview, in order to verify enough deepening and the internal logic of the object of study in the light of the proposed framework.

### Selection criteria

Mothers, fathers, or guardians of transgender adolescents that had access to the scheduling link provided along with the research description were included. People who showed impediments or limitations to participate during the period of data collection were excluded. In the scheduling stage, there were four waivers of participation in the interview, whose exclusion occurred after three attempts to contact the participants to reschedule.

### Participants

Participants were selected using the snowball technique, a variant of convenience sampling, in order to gather the population of interest that is difficult to access due to social stigmas^24^ ^-^ ^25^.

In accordance with the snowball technique, contact was initiated with professionals or representatives of the study sites, who were the “seeds”. On that occasion, the objective of the study was explained, and the strategies were adapted to collect data in accord with the dynamics of the place, aiming to share the scheduling forms with possible participants.

The indicated people composed the zero wave, and were part of the sample. Given the difficulty in accessing this target group, these people also had the function of indicating other participants in order to compose the subsequent waves.

### Instruments used to collect information

The instrument of characterization and script of the semi-structured interview was elaborated by the researcher, and its contents were reviewed by the expertise of a research group. The instrument was subjected to a pre-test, conducted with the first eight participants of the study, who self-filled the instrument of data collection.

The application revealed answers in line with the open questions, however, with little deepening for a satisfactory analysis of the investigated phenomenon, revealing the importance of the interaction between researcher and participant when conducting qualitative research, which includes observations of the emotion expression through gestures, beyond speech.

### Data collection

Due to the geographic distance of some participants, the context of the COVID-19 pandemic, and the limitations found in the self-filling of the data collection instrument, individual interviews were carried out remotely and face-to-face using the Google Meet tool. It is important to highlight that the participants of the pre-test were not included in the study, with the exception of two, who, in a second opportunity of scheduling, were interviewed face-to-face.

In this way, after the pre-test, the collection occurred as follows: first, an access link to a Google Forms form was provided at the research places, along with the description of the research, which allowed the scheduling of interviews with possible participants of the study, seeking to adapt to their availability and protect an ambience that allowed an in-depth verbalization, especially regarding questions that involved social stigmas.

On the arranged day and time, participants received a link to access the Google Forms form with the Free and Informed Consent Term (TCLE), and closed questions for the purpose of characterizing the study population. Then, participants received a link to access Google Meet for the individual interviews, which were conducted by the researcher herself, who is experienced in qualitative research, and by a previously trained member of the research group. At the end of the interviews, participants were invited to indicate other potential participants. It is important to emphasize that filling out the characterization instrument, and participating in individual interviews lasted, on average, 60 minutes.

### Data processing and analysis

The individual interviews were audio/video-recorded and, subsequently, fully transcribed. The textual *corpus* was subjected to Content Analysis in the thematic modality^26^. The first stage consisted of the pre-analysis with fluctuating reading of the empirical material, in addition to consideration of the rules of exhaustiveness, representativeness, homogeneity, pertinence, and exclusivity. The second stage involved the exploration of the material: data coding and semantic aggregation of the words, while the third stage comprised the results processing in an interpretive way for nominating the categories in the light of the theoretical framework^26^.

### Ethical aspects

This study followed Resolution no.466/12^27^. After the consent of the research sites, the project was submitted and approved by the Research Ethics Committee of the Ribeirão Preto College of Nursing (CEP-EERP/USP), according to report no. 4,567,837. The CEP-EERP/USP sent the CEP a copy of the two co-participating institutions that had their own committee, one located in the northeast region (report no. 4,759,691), and the other in the southern region of the country (report no. 4,655,270). The consent of participants was given through the TCLE.

The names of the participants were replaced by names of flowers or plants, suggested by the researcher, and, later, chosen and/or authorized by the participant, with substitutions and requests for the permanence of the proper name being authorized.

## Results

In this study, 22 guardians of transgender adolescents were included, being 20 mothers and two fathers, from the states of Pernambuco, São Paulo, Rio de Janeiro, Espírito Santo, Goiás, Minas Gerais, and Rio Grande do Sul.

Among the participants, all declared themselves to be cisgender, 20 reported being heterosexual, one homosexual, and one bisexual. The average age of the participants was 46 years old, therefore, adults. In relation to marital status, 10 declared themselves to be married, seven single, two separated, two divorced, and one in a stable union. Regarding education, eight graduated from Graduate School, eight graduated from College, three did not finish College, two graduated from High School, and one did not finish Technical School. Concerning income: four earned more than 6 minimum wages (MW), six earned between 5-6 MW, seven earned between 3-4 MW, four earned 1-2 MW, and one earned less than 1 MW.

### Access to qualified services and support in the health care network

The guardians of transgender adolescents reported having difficulty in accessing specialized health services capable of monitoring from childhood to adolescence. It is also possible to verify the lack of preparation of health professionals on the subject:

*So, this multidisciplinary team I mentioned, that the resolution of the Brazilian Federal Council of Medicine launched, in Brazil we only have three outpatient* clinics, let’s say, capable of caring for children *[...] So, there are three cities that deal with a lot of people? Yes. But Brazil is very big, is it a first step? Yes, but I mean, there should be more care, more support, ok? More decentralized* (Saint George’s sword).

*[...] They have to mobilize from different places of Brazil with air ticket, accommodation, to come and see the kids here in [State], you know? It’s an absurdity, Brazil is huge, there must be this everywhere, every city must have care, every State, every capital, it’s* outrageous not to have it (Rose).

*[...] the hospitals should expand… it is not enough in relation to the amount of people who seek [...] I chose private health care because I could not find in the public service [...] a nurse, who interviewed us, said it would be no longer possible to participate in the hospital, public service, right? But she recommended someone to me. Even when choosing private health care, it isn’*t easy. *It’*s not a matter of money, but a matter of who could provide this service, even in private health care? (Dove orchid).

*There is no outpatient* clinic that deals with that in the private network. *Private doctors who provide this kind of care charge whatever amount they want. So… the family was wondering if they would need an indebtedness to be able to advise my daughter… a much larger network is needed… and the outpatient* clinic was fundamental, otherwise I wouldn’t have been able to find an endocrinologist until now… it took me more than a year to find one, just so he could check my daughter’s hormones *[...] this is an absurdity, this is prejudice, this is transphobia, but it happened to me* (Rose).

*The first integral health care outpatient clinic for transgender people has been recently inaugurated in my city, but it’s only for those who are over sixteen, so the [son] can’t use it yet, but I think that’s it, like, if the professionals were trained, if the health network in general was prepared to receive trans people, there would be no need for an outpatient clinic* (Azalea)*.*

*We haven’t been able to move to a specific outpatient clinic yet. We can’t find that here in my city (hormonization). There should be an outpatient clinic in the city that has a specific follow-up for them* (Hydrangea).

*Well, we don’t, right? [specialized follow-up], now I’m going to visit the [specialized outpatient clinic], I hope it’s good. I have a lot of expectations, I’m even holding mine, because it’s my hope here, we have the [specialized outpatient clinic from another state], I’m on the waiting list, which is huge […] first of all, it makes me happy that it will be part of the SUS, because I believe so much in the SUS, and I want it to have this psychological, psychiatric, and endocrinological support in the SUS for life* (Bromelia).

Other participants emphasized the lack of specialized service in the municipality, and the need to seek care in a health service belonging to a distant municipality for the purpose of emotional and informational support, as well as health monitoring for transgender adolescents.

*The queues today are too long, all with more than a year of waiting […] all the anxiety [of the kids] is very big, and when they say […] we can’t stand it anymore. The [hospital] in [municipality where they live] does not have multidisciplinary follow-up, just a gynecologist… we are being monitored by a multidisciplinary team at the [referral hospital in another municipality]. The team has psychiatry, child and adolescent psychiatry, psychology, nursing, speech therapy, pediatrics, endocrinology, gynecology, art therapy, and anthropology* (Saint George’s sword).

*[…] we registered her there [specialized service], it’s been more than two years, nobody calls, nobody says anything […] it’s basically zero, especially the SUS […] when she went to the health center, the doctor who attended was very thoughtful and referred her to an endocrinologist, he indicated only the adult service, right? Who authorizes it, denied it, said she didn’t need it, that the SUS wouldn’t authorize it* (Orchid)*.*

This scenario worsened during the COVID-19 pandemic, with the restriction of support for transgender adolescents and their guardians in the context of the SUS:

*I spent a year struggling to find free care, because I couldn’t afford paid care. The pandemic suspended services, so when it completed one year […] we managed to start using public care […] I got a health care plan, because I know we need it, everything was very difficult through the SUS […] give up something to be able to pay for a health care plan* (Lily)*.*

*The lack of access to public services, and of support by qualified professionals can lead to health risks, resulting from indiscriminate hormonization, as observed in the following reports:*

*As for health, about her doing hormonization, there is this trans woman who is part of our association, and she is an endocrinologist, graduated from the Federal University of our State […] we can’t afford it. We registered her through the SUS, it’s been more than two years, and nothing, nobody calls […] and regarding the psychological treatment […] we also attend to the group of mothers, using the SUS is very complicated, that’s why so many people end up doing everything on their own, which results in so many serious consequences related to it, hormonization […]* (Orchid)*.*

*[…] I wanted this guidance [on hormonization] from someone who works with adolescent health. I need to talk to someone who understands about it. I have a cousin who is an endocrinologist, but she doesn’t work in this area. We don’t have much access, so we don’t really know what to do, right? I’m pretty lost, you know?* (Jade plant)*.*

*Concerning the lack of qualified specialists in the municipality, and the resistance to referrals, one of the mothers reported difficult and bureaucratic experiences:*

*[…] we registered in the outpatient gender identity clinic of the [University], and managed to go through triage there […] I’m solving the bureaucratic problems, because I’d have to get a bus ticket to go there with him. I recently got support here from within the primary care center in my neighborhood […] we saw a doctor who is from our sector, she called this trans doctor who welcomed us very well and referred us to another outpatient clinic, she guided us in the best way possible* (Sunflower)*.*

### Visibility: the look designed to support in the health field

The guardians of transgender adolescents emphasized the importance of acceptance, emotional and informational support, and ambience, based on the reports below:

*The more information, the less we suffer. It’s not as if it will solve everything, but we suffer a little less, because we can anticipate and understand the things that happen and we see that we’re not alone, you know?* (Saint George’s sword).

*I think we need the informational (support), even in terms of health, you know. Nobody tells you […] do that, it’s the mothers who say, but in health, if you look for the doctor: “I don’t even know her’’. People at the very outpatient clinic I went to didn’t know there was a place that provided care* (Alstroemeria).

*[…] I think the health institutions (should support) […] it should be a more normal thing. Since it is health, for example, there is the campaign for women […] there should also be support for transgender adolescents* (Heliconia).

*I think all kinds of information, mainly for children, about school, about how to deal with, or how to talk about the use of bathrooms, about documents, about health, and I mean, the most important thing, besides the material, would be a support, a service that can be provided here* (Saint George’s sword).

*[…] in a primary care center, all the posters, there’s only cis-hetero representativeness, it is always a man, a woman, a white child, one of those. No LGBT population, right? He arrives there, never feeling welcome, and has to go to the gynecologist, to the ophthalmologist, and never has any referrals, you always “can’t talk’’, I think it should happen in public spaces* (Tulip).

*[…] the professionals themselves don’t know what to do, right? There is no policy, at least it’s not mentioned much […] If I go to the family health center here, they won’t know it exists, not will the schools […] it’s something that needs to be promoted, said, because there may be lots of kids suffering, adolescents suffering, and they don’t know why* (Alstroemeria).

*There is a lack of training, this showing of duty is only mentioned in law […] but the law only comes if the duty is fulfilled, and the duty of the professional is precisely that, to remember that they are an institution. Nowadays we value a lot the matter of empathy, right?* Something that not everybody understands, that we always need to teach, but today we see it is the law, right? *Something that you can’t simply disrespect. It’s the law. If you don’t obey, it’s considered a crime in many cases* (Laelia).

*The law doesn’t guarantee that things will happen, right? Even an older person, for example, who has been working with the population for thirty years, it’s not that they are against it and think these people are some kind of aberration or anything like that, but because they don’t even know what it’s all about* (Alpinia).

Within the community, health promotion in schools is an important strategy for visibility and support, however, about the context of school, it was reported:

*[…] he was persecuted by three classmates inside the school, and the institution did not support him, and that was when his mother and I became aware, because his performance started to drop drastically […] he was being bullied and the school did not do anything, so we had a very big fight against the school, transferred him to another school, and after that we sought and found a psychologist for him […]* (Mandacaru)*.*

*Regarding the spaces that provide some kind of assistance, it was mentioned:*

*[…] I get a lot of information from there [Non-governmental Organization (NGO)], they are mothers who helped me a lot! Sometimes I say something and ‘’oh, I’m sorry, I don’t know if I’m saying it right’’, they say ‘’relax, you may say, and if it’s not right, we’ll kindly explain to you: ‘’so, that’s not how we say’’.*(Jasmine)*.*

*[…] they are civil society initiatives, but the government doesn’t have this information, which is so clear to the population, and I still feel like I’m in a privileged place, because I am part of a group for diversity, I can get access, I have internet, anyway, there people who are not in this situation* (Poppy)*.*

*In the group, we also exchange a lot of experiences and information about laws, for example, we share many laws because there are Federal and State laws, and that was how I discovered things I got here in [State of residence] very easily, some mothers hadn’t got them yet* (Fleur-de-lis)*.*

*The speeches revealed the lack of visibility and support of potential spaces for health promotion:*

*I was not informed of anything in the health center we have here. The doctors seem to not know anything […]. Okay, my daughter is transgender, now what? Without this answer we are alone, we have to look for it to know, to find out by ourselves […] There is no path* (Cactus)*.*

*There are groups [in the municipality] that is the LGBT forum, and there is also a group of trans, travestis, but that is more related to adults […] there are groups, but nothing related to health, for example, there is no LGBT council here, there would have to be more qualified professionals for that, outpatient clinics, and support, both for children and adolescents, as well as for parents* (Amaryllis)*.*

*It doesn’t reach the media, we don’t have access, because after you enter the groups, then you belong to these places, but until I got there, I met people who helped me and people who put obstacles in the way of this process* (Tulip)*.*

*[…] After a lot of research, and even in the service we saw that they didn’t offer that, it was when the opportunity appeared through the [NGO], a live, the people there [from the specialized outpatient clinic], the doctors in charge provided the opportunity, then we registered, I think it took us around six months, when they called* (Dahlia)*.*

## Discussion

Even though the SUS Transsexualization Process is an important achievement of social movements for universal of access, this doctrinal principle is below the coverage of most transgender people. In Brazil, the scarce referral centers for monitoring transgender children and adolescents are centralized in the southeastern and southern regions of the country^16^, in which hormonization can be started at puberty exclusively in experimental character of research protocols, in accord with the norms of the CEP/Conep System^17^.

The acceptance of transgender children/adolescents and their families need to be implemented since the first years of life, as gender identity can be recognized from the age of two^9^. However, the findings of this study indicate important barriers to access, due to long waiting lists for specialized services, that can last for years, resulting in suffering, especially in the first phase of adolescence, due to the late or non-existent approach by health services^3^. This scenario leads to reflections both regarding the need for expansion and decentralization of these specialized services in the national territory, but also concerning the strengthening of the health network, through the training of health professionals who work both in PHC and in specialized care, and the perspective of strengthening intersectoral articulation.

Barriers to access to health services constitute care voids, permeated by the nonexistence of referral and counter-referral in the health care network, and difficulties of support in the municipalities of residence, with the search for alternative, yet costly, solutions in private services. In addition, this population can seek help in voluntary services connected with the third sector, or even in unofficial places, whose clandestinity may confer a high potential risk to the transgender adolescent health.

In this study, the participants reported the importance and urgency of a health policy in a network, which envisages the provision of actions and services capable of enabling access to care, in addition to overcoming potential economic barriers. Despite the existence of specific and transversal policies in the national scenario, the statements reflect not only the lack of knowledge on the part of the participants, but, above all, they reveal the weaknesses in the visibility and concreteness of such politics in the context of social and health practices.

Another identified aspect that confirms other studies^1^ ^-^ ^4^ ^)^ refers to the fact that the doubts are not always clarified by health teams, which reveals the need for strengthening a gateway, as well as the definition and recognition/legitimacy of a support itinerary defined by professionals for the transgender adolescents care in the context of the HCN, through a network of care that organizes the flow and gives support to transgenderism in the children and youth period.

In the context of the COVID-19 pandemic, the participants of this study indicated that the transgender adolescent health care was even more precarious, perceived as less important, whose demands were configured as selective and not urgent, contributing to greater emotional pain. It is important to emphasize that the beginning of puberty is a complex phase, which demands support, especially to those transgender adolescents that express a desire for body changes. In addition to the indiscriminate hormonization, industrial silicone, breast bands, binders, and other methods are frequently used without the recommendation of health professionals^10^ ^,^ ^17^.

Even in developed countries, such as Canada, a study performed with parents of transgender adolescents revealed a long waiting time for the beginning of hormonization, and lack of therapists for their children, which generated greater tension^28^. These limitations result in anguish for parents, resulting from the potential emotional pain of their children, due to the emergence of sexual characteristics contrary to their gender identity, caused by the delay of hormonization and possible limitation of late results^29^.

Studies conducted in Canada and the United States emphasized the importance of recognizing the gender identity of transgender children, which occurred among their guardians, who were committed to their well-being in order to protect them from bullying, depression, anxiety, self-harm, and suicide^30^ ^-^ ^32^. In this sense, the support of professionals can help in the search for ‘’solutions’’ that these guardians present^33^.

In relation to transphobia in the school environment, Brazilian transgender adolescents face limitations to use bathrooms, in addition to physical and psychological aggression, situations of family imposition in the choice of their clothing, labels, lack of understanding by society, and invasion of their intimacy. In this sense, the dialogue with the adolescent and guardian aims at the knowledge and understanding of their demands, without exposing or invading their intimacy, let alone making them feel weird^9^. For this purpose, the participants of this study proposed greater visibility of the theme, including in schools, since it allows for greater extramural actions in the territory through the School Health Program, and community awareness to face transphobia

Acceptance and qualified listening are technologies capable of guiding care from the perspective of trans citizenship^18^, through motivation and support for active participation in the health care production process, however, gender diversity is still conducted by some professionals as a pathology^3^ ^,^ ^10^.

A study carried out in Italy revealed that guidance on transgenderism was given from parents to health professionals, whose experience culminated in conflicts between them; besides, the specialized services were located essentially in hospitals, in such a way that ambience referred to the pathological perspective^34^. Ambience was reported by one of the participants of this study as an important resource for belonging to public health spaces.

Regarding parents or guardians, the mother figure is considered the first person that adolescents seek to start a dialogue about the recognition of their gender identity and for support^9^, which is consistent with the profile of the participants in this study, who were mostly mothers.

Moreover, this research points out important weaknesses of the participants in dealing with structural transphobia. In this direction, other studies also reported isolation from parents/guardians, feeling of fear, rejection, guilt, ambiguous loss of the idealized child (cisgender), lack of knowledge, and need for support from their social network^35^.

The participants of this and another study^9^ ^)^ also mentioned that the support from health professionals, emotional and informational, and from other parents (in-person and online), contributed to the understanding of their children’s situation and their role. On the other hand, other parents stated that they felt excluded from decision-making processes, and some described communication problems with health professionals and denial of the timely access to necessary care.

In this study, only one participant mentioned Nursing, showing the gap in access to trans-specific care provided by nurses, and the lack of understanding by civil society about the potentialities of the profession. This situation can be explained by a study that revealed a lack of knowledge on the part of nurses working in PHC, with behaviors in line with the biomedical training, and lack of skill to meet the needs of transgender people, whose actions are still conducted in a binary way. Such elements can contribute to the weakening of bonds in a broad and complex context, permeated by structural dynamics that sediment transphobic violence, whose confrontation demands transformations, especially in the training of human resources. In this direction, it is worth mentioning the need to review the curricular matrices of the pedagogical projects of Nursing courses, in order for them to include integral health care, from the perspective of care for sexual and gender diversities^36^.

Still with regard to integral care provided by Nursing, it is noteworthy that transgender people have general health demands, and some specificities related to their needs, requiring professional skills for support, overcoming pathologizing views and approaches, advancing towards recognition and naturalization of a perspective of being and existing. In this sense, the training of nurses, in their course curriculum, has the potential to transgenderism based on disciplines of policies, management, and life cycles in the health care production process, including the recognition of gender identity issues, even when they are not the main demand, but configured as transversal demands with a view to a trans-specific network of care.

Regarding the potentialities for expanding the clinical practice of nursing with transgender adolescents in PHC, there are opportunities for the protagonism in clarifying doubts and demystifying social taboos, rights to the social name, and policies in the context of health, as well as the link between peer networks for continuous support, referral to qualified services according to the demands, recommendations and implications related to hormonization, and other resources for feminization or masculinization.

Furthermore, aspects related to sexual and reproductive health, and inclusion in cancer prevention actions; fight against transphobia; family support for decision-making empowerment; team qualification, including doormen, attendants, community health agents, and other team professionals; adjustment of vaccination record to the gender identity that is still binary and cisgender; promotion of ambience and advocacy; participation in discussions with the interdisciplinary team for the STP construction; participation in courses promoted by management and specializations for qualification in conduct at different health care network points, including medium and high complexity, are pointed out.

When thinking about the logic of continuous and coordinated care in the context of a health care network, the prerogative is the strengthening of PHC, especially the FHS as a priority model, since it has a fundamental role in providing decentralized, equitable, and longitudinal actions in the care provided to transgender adolescents and their social network since childhood. The potential of PHC through the STP is reiterated as a care technology centered on the person/family, in addition to the promotion of collective and emancipatory actions, articulated with trans-centered networks, with the purpose of promoting health education and mutual support between pairs. In this sense, advancing in the proposition, implementation, and evaluation of individual and collective actions that meet complex needs and demands of transgender adolescents and their parents/guardians requires synergism in the team performance, and it is essential to value matrix support, which is weakened in the current scenario of PHC funding policies and FHSC incentives^37^.

This research advances in the visibility of challenges that involve the accepting and listening to mothers, fathers, and guardians of transgender adolescents, as the first network of socialization, for the purpose of promoting the necessary support in such a way that they feel able to support their children since childhood, with a view to integral health care. Moreover, it reiterates the insufficiency of qualified services to approach transgenderism in childhood/adolescence, and the need for decentralization of actions and services to favor timely and equitable access, demanding articulation and political mobilization, as well as nursing qualification for the health team, in the context of the health care network.

In the face of this broad and complex scenario, the potential of Nursing, articulated to the multi/interdisciplinary team, in the support of transgender children and youth, through spaces and opportunities to stimulate the protagonism of adolescents and their families/guardians, stands out.

The production and management of health care for transgender adolescents articulates interrelated actions in the exercise of Nursing under individual, family, professional, organizational, systemic, and societal dimensions^21^. In this sense, it contemplates the promotion of trans autonomy in self-care, as well as the support for the nuclear family and other actors in the social network for empowerment and support for transgenderism. It also demands the qualification of nurses for support, in addition to agendas, protocols, team meetings, planning, evaluation with an emphasis on trans citizenship, construction of support flows through the elaboration and operationalization of networks of care that focus on gender diversity, implementation of social and health policies, accountability of the State, and mobilization of organized civil society in social participation.

It is important to highlight the focus of this study, anchored in the perspective of parents/guardians, however, this listening does not waive or replace the speech of their children, on the contrary, they complement each other in the intense process of identifying the needs of both.

In view of these findings, it is recommended an investment in new studies that address the listening to trans adolescents in different social contexts, health professionals working in multi and interdisciplinary teams, educators, managers, and social leaders with the purpose of identifying gaps when guaranteeing the rights of trans adolescents, and subsidizing the proposition of actions to fight against structural transphobia.

The limitation of this study emerged due to the fact that the participants joined support networks of organized civil society, which may have suppressed the experience of those who did not have access to the research, or those who do not recognize the gender identity of their children.

## Conclusion

Integral health care for transgender adolescents from the perspective of their parents/guardians reveals the lack of support and the centralization of care in scarce specialized units, with the need to share health actions and services in the context of the network, with an emphasis on the FHS valorization as a gateway, in addition to the proposition of a trans-specific network of care, and production/propagation of protocols/educational materials with a view to trans citizenship.

This research indicated as a prerogative the importance of the joint and coordinated performance of the multi and interdisciplinary team, with the proactivity of the nurse in producing and managing health care for transgender adolescents and their parents/guardians, through the provision of individual and collective actions, health promotion, including articulation with schools for visibility and support, ambience, and awareness to trans demands since childhood, in order to mitigate possible psychological pain and health risks.

The nurse’s role demand empathy, support, and active listening to the other, so that they can express who they are, what their experience is, what their needs are, and how qualified professional work should be conducted. Professional behaviors should not be associated with prejudiced moral precepts, since recognizing gender diversity and variability is an ethical commitment to legitimize existence, guaranteeing rights and defending life.
